# Malectin Domain Protein Kinase (MDPK) Promotes Rice Resistance to Sheath Blight via IDD12, IDD13, and IDD14

**DOI:** 10.3390/ijms23158214

**Published:** 2022-07-26

**Authors:** Zhibo Cui, Caiyun Xue, Qiong Mei, Yuanhu Xuan

**Affiliations:** 1 College of Plant Protection, Shenyang Agricultural University, Shenyang 110866, China; cuizb@syau.edu.cn (Z.C.); syauxue@syau.edu.cn (C.X.); 2 Rice Research Institute, Shenyang Agricultural University, Shenyang 110866, China

**Keywords:** malectin domain protein kinase, sheath blight, resistance, IDD, rice

## Abstract

Sheath blight (ShB) caused by *Rhizoctonia solani* is a major disease of rice, seriously affecting yield; however, the molecular defense mechanism against ShB remains unclear. A previous transcriptome analysis of rice identified that *R. solani* inoculation significantly induced *MDPK*. Genetic studies using *MDPK RNAi* and overexpressing plants identified that *MDPK* positively regulates ShB resistance. This MDPK protein was found localized in the endoplasmic reticulum (ER) and Golgi apparatus. Yeast one-hybrid assay, electrophoresis mobility shift assay (EMSA), and chromatin immunoprecipitation (ChIP) showed that the intermediate domain proteins IDD12, IDD13, and IDD14 bind to the *MDPK* promoter. Moreover, IDD14 was found to interact with IDD12 and IDD13 to form a transcription complex to activate *MDPK* expression. The three IDDs demonstrated an additive effect on *MDPK* activation. Further genetic studies showed that the *IDD13* and *IDD14* single mutants were more susceptible to ShB but not *IDD12*, while *IDD12*, *IDD13*, and *IDD14* overexpressing plants were less susceptible than the wild-type plants. The *IDD12*, *IDD13*, and *IDD14* mutants also proved the additive effect of the three *IDDs* on *MDPK* expression, which regulates ShB resistance in rice. Notably, *MDPK* overexpression maintained normal yield levels in rice. Thus, our study proves that IDD12, IDD13, and IDD14 activate *MDPK* to enhance ShB resistance in rice. These results improve our knowledge of rice defense mechanisms and provide a valuable marker for resistance breeding.

## 1. Introduction

The susceptibility of rice to various diseases adversely affects yield production worldwide [1]. ShB caused by the hemibiotrophic pathogen *Rhizoctonia solani* Kühn is a highly destructive disease, which significantly threatens rice cultivation. ShB resistance is considered a quantitative trait controlled by multiple genes [2]. Since 1995, many quantitative trait loci (QTL) for ShB resistance have been mapped and functionally characterized [3]. Subsequently, breeders have generated resistant varieties by polymerizing the QTL associated with disease resistance and introducing them into cultivated rice [4]. However, no dominant ShB resistance gene has been identified in the natural rice populations, which challenges breeding for disease resistance [5]. 

Modern genetic engineering methods and genome-wide association studies (GWAS) of natural populations have been used for identifying plant defense-related genes. For example, the F-box protein ZmFBL41 was characterized in the resistance to banded leaf and ShB in maize [6]. The resistance protein RPM1 encoded by *OsRSR1* and the protein kinase gene *OsRLCK5* are newly confirmed to positively regulate ShB in rice [7]. The study of *OsNYC3*, a chlorophyll degradation gene, demonstrated that chlorophyll content is positively correlated with rice resistance to ShB and improves yield [8]. Additionally, the specific introduction of foreign genes such as *AtNPR1*, *BjNPR1*, and *ZmPGIP3* enhanced ShB resistance and yield in rice [9,10,11]. In parallel, the tissue-specific activation of DOF11 fused with VP16 increased both yield and ShB resistance [12]. These studies have indicated the possibility of a balance between yield and disease resistance. 

Furthermore, the exploration of defense mechanisms against ShB is a crucial issue. The tau class of glutathione-S-transferase in rice, OsGSTU5, is an important defense-related protein that improves disease resistance by resisting reactive oxygen species (ROS) accumulation [13]. The protein phosphatase 2A catalytic subunit, OsPP2A-1, positively regulates defense gene expression and enhances ShB resistance [14]. The overexpression of *OsCHI11*, *OsWRKY30*, *OsACS2*, *OsASR2*, and *LPA1/IDD14* enhanced resistance to ShB [15,16,17,18,19]. Furthermore, the intermediate domain proteins such as IDD13 and IDD3 have been shown to interact with LPA1, thereby positively and negatively regulating rice resistance to ShB, respectively [20]. The DEP1 negatively regulates ShB defense by interacting with LPA1 and inhibiting its DNA binding activity [21]. RAVL1 activates ethylene and brassinosteroid (BR) signaling and *IDD3* to modulate rice resistance to ShB [22,23]. Recently, kinesin-like protein KLP was reported to interact with LPA1 to promote ShB resistance [24]. These studies suggested IDD proteins play important roles in ShB resistance. In addition, the indeterminate domain (IDD) proteins play various biological functions in plants. IDD is composed of four zinc-finger (two C_2_H_2_ and two C_2_HC) motifs. In rice, 14 IDD proteins have been identified, which contain either the MSATALLQKAA or TR/LDFLG conserved domains, or both at the C-terminus regions of IDD peptides [25]. IDD proteins act as important regulators in plant development and responses to environmental factors. They play crucial roles in secondary cell wall formation [26], stem elongation [27], shoot gravitropism [28], chilling tolerance [29], floral transition [30], and ShB resistance of rice [20,21]. However, the targets of IDDs are largely unknown. 

Malectin is a membrane-anchored protein of the endoplasmic reticulum that recognizes and binds with Glc2-N-glycan; the domain is found on many plant receptor kinases. Recent studies on the malectin domain proteins focused on malectin domain receptor-like kinases (RLKs). *Required for non-host resistance 8* (*Rnr8*), encoding HvLEMK1, an LRR-malectin domain-containing transmembrane RLK, regulates non-host resistance of barley to *Blumeria graminis* f.sp. *tritii* [31]. Meanwhile, LETUM1, a malectin-like RLK, regulates autoimmunity in *Arabidopsis* by interacting with SUMM2 and MEKK2 and forming a complex [32]. Malectin/malectin-like domain proteins play important roles in other physiological processes, such as pollen and seed development, growth, disease resistance, and survival [33,34,35,36,37]. Moreover, we identified that *R. solani* significantly induced *MDPK* [38]. Nevertheless, malectin domain kinase’s function and mechanism in regulating ShB resistance have not been reported. 

Therefore, the present study assessed the interaction between the indeterminate domain (IDD) protein family transcript factors IDD12/13/14 with the promoter of *MDPK*. Various assays, namely the yeast two-hybrid assay (Y2H), bimolecular fluorescence complementation (BiFC), and coimmunoprecipitation (Co-IP), were used to analyze these interactions. Further gene function studies were performed in plants to explore the role of MDPK and IDDs in rice defense. Collectively, the present study shows that IDD12, IDD13, and IDD14 impose an additive effect on *MDPK* activation and positively regulate ShB resistance in rice. 

## 2. Results

### 2.1. MDPK Positively Regulates Rice Resistance to ShB

**Figure 1:**
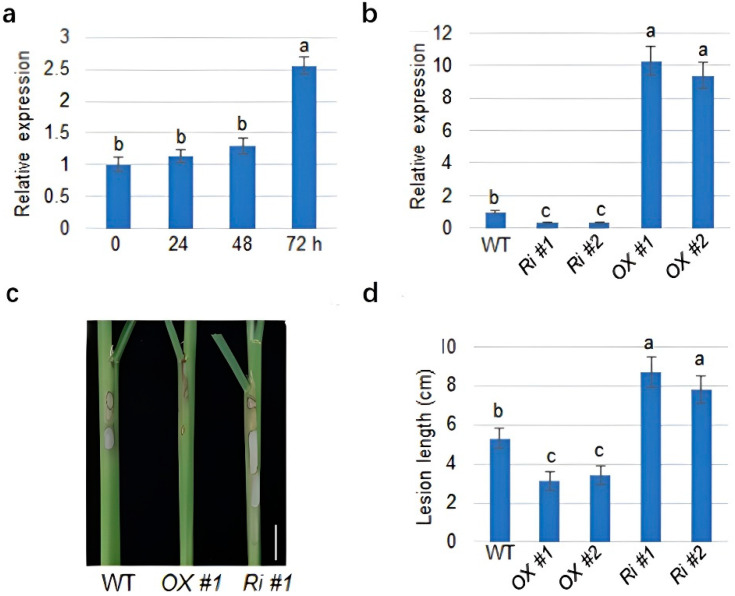
The regulation of *MDPK* to ShB resistance: (**a**) relative expression of *MDPK* in wild-type (WT) at 0, 24, 48, and 72 h after inoculation with *R. solani* AG1-IA. The error bars mean ± SE (*n* = 3); (**b**) the expression level of *MDPK* in wild-type, *MDPK RNAi* (*Ri #1*, *Ri #2*), and *MDPK* overexpressing (*OX #1*, *OX#2*) plants. The error bars mean ± SE (*n* = 3); (**c**) the response of *MDPK* RNAi plants (*Ri #1*) and *MDPK* overexpressing plants (*OX #1*) to *R. solani* AG1-IA compared with the wild-type. Each experiment was performed in triplicate; (**d**) the lesion lengths on sheath shown in (**c**) were calculated. Data indicate average ± standard error (SE) (*n* > 10). Bonferroni’s multiple comparison test, was used for the calculation of comparisons between different groups. Letter a, b and c in the figure indicate significant differences at *p* < 0.05.

Rice plants infected with *R. solani* rapidly reprogram the transcriptome to defend against the infection [39]. *Malectin Domain Protein Kinase* (*MDPK*, *LOC_Os09g18594*) was one of the several genes induced 72 h after *R. solani* infection (Figure 1a). *MDPK* homolog gene was shown to play a key role in arbuscular mycorrhizal (AM) symbioses in rice [40], implying that this type of gene may play roles in plant and microbe interaction. Therefore, to analyze the function of *MDPK* during infection, RNAi and overexpressing plants were generated in this study. The qRT-PCR analysis confirmed lower *MDPK* expression levels in the *RNAi* (*Ri*) lines and higher expression levels in the overexpressing (*OX*) lines compared with wild-type plants (Figure 1b). Interestingly, the inoculation of *R. solani* AG1-IA in these plants showed that the *MDPK RNAi* plants were more susceptible, while the *MDPK OX* plants were less susceptible than the wild-type (Figure 1c,d). 

### 2.2. IDD12, IDD13, and IDD14 Activate MDPK Transcription 

**Figure 2:**
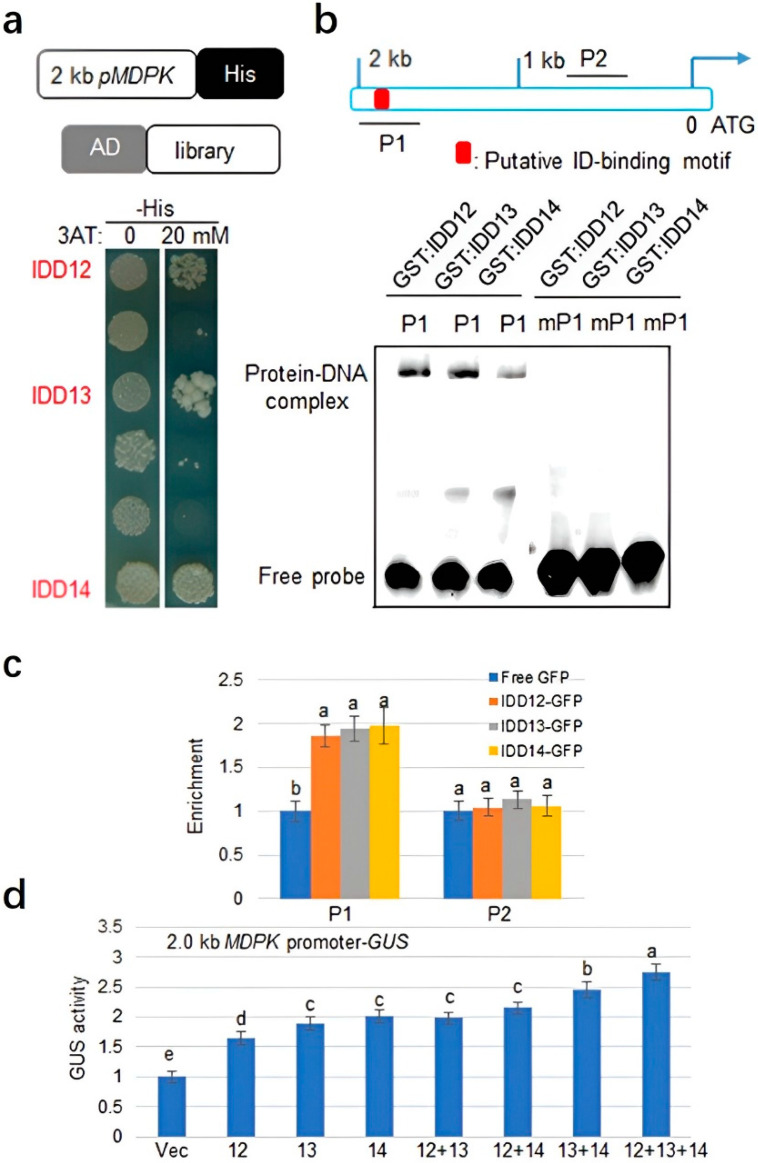
The regulation of *IDD12, IDD13*, and *IDD14 to MDPK* transcription: (**a**) yeast one-hybrid assay revealed that IDD12, IDD13, and IDD14 can bind to the promoter of *MDPK*. The upper schematic indicates the 2 kb of *MDPK* promoter and rice cDNA library used for the yeast-one hybrid analysis. The transformants were grown on the SD media with or without 20 mM of 3-amino-1,2,4-triazole (3AT), a competitive inhibitor of HIS3; (**b**) EMSA assay showed that IDD12, IDD13, and IDD14 have specific affinity to the putative IDD-binding motif (red box) P1 within 2 kb of *MDPK* promoter. The mutant probe mP1 was used as control. The probe was labeled with biotin and bands were detected by using antibiotin antibodies; (**c**) ChIP-qPCR assays. Enrichment of IDD12, IDD13, and IDD14 at the promoter of *MDPK* was measured via ChIP-qPCR using anti-GFP antibodies. The schematic in (**b**) indicates the locations of the DNA fragments P1 and P2 used for ChIP-qPCR. Error bars represent ± SE (*n* = 3); (**d**) a transient expression assay was conducted by cotransfection with empty vector (Vec), *p35S:IDD12* (12), *p35S:IDD13* (13), *p35S:IDD14* (14), *p35S:IDD12* + *p35S:IDD13* (12 + 13), *p35S:IDD12* + *p35S:IDD14* (12 + 14), *p35S:IDD13 + p35S:IDD14* (13 + 14), or *p35S:IDD12* + *p35S:IDD13 + p35S:IDD14* (12 + 13 + 14) and the reporter vector expressing the *beta-glucuronidase* (*GUS*) under the control of 2 kb *MDPK* promoter. The luciferase gene driven by the 35S promoter was used as an internal control to normalize GUS expression. Error bars represent ± SE (*n* = 3). Bonferroni’s multiple comparison test, was used for the calculation of comparisons between different groups. Letter a–e in the figure indicate significant differences at *p* < 0.05.

*Rhizoctonia solani* induced *MDPK* expression levels. Therefore, the study aimed to identify the upstream regulators of *MDPK* using a 2 kb region of the promoter and a rice cDNA library. The Y1H assay showed that the IDD transcription factors IDD12, IDD13, and IDD14 bind to the 2 kb region of the *MDPK* promoter (Figure 2a). Subsequent sequence analysis indicated that an IDD-binding motif was located within the 2 kb of the *MDPK* promoter. Further, an EMSA was performed to verify the binding of three IDD proteins to the *MDPK* promoter. Here, GST-IDD12, GST-IDD13, and GST-IDD14 were bound to the putative IDD-binding motif P1 but not with the mutated probe mP1 in vitro (Figure 2b). Moreover, the binding activities of IDD12, IDD13, and IDD14 at two regions on the promoter of *MDPK* were analyzed via chromatin immunoprecipitation (ChIP) assay. In the *35S:GFP* and *35S:IDD:GFP* transgenic plant calli, the amplicons of P1 are highly enriched, which are the *MDPK* transcriptional start site containing the putative IDD-binding motif. This means IDD12, IDD13, and IDD14 were bound to P1 but not P2 (Figure 2c). In addition, transient expression assays were performed via the cotransformation of the *MDPK* promoter and a combination of *35S:IDD* plasmids and a vector expressing the beta-glucuronidase gene (GUS) under the control of pMDPK. IDD13 and IDD14 exhibited higher transcription activation activity than IDD12. Moreover, IDD13 + IDD12 or IDD14 + IDD12 coexpression showed similar effects on *MDPK* promoter activation compared with IDD13 or IDD14, respectively, but IDD13 + IDD14 coexpression demonstrated a more robust transcription activation activity than the expression IDD13 or IDD14 alone. Moreover, the coexpression of IDD12, IDD13, and IDD14 showed the strongest transcription activity to *MDPK* promoter compared with IDD13 + IDD12, IDD14 + IDD12, or IDD13 + IDD14 coexpression groups, indicating an additive effect of IDD12, IDD13, and IDD14 on *MDPK* transcriptional activation (Figure 2d). 

### 2.3. IDD14 Interacts with IDD12 and IDD13

**Figure 3:**
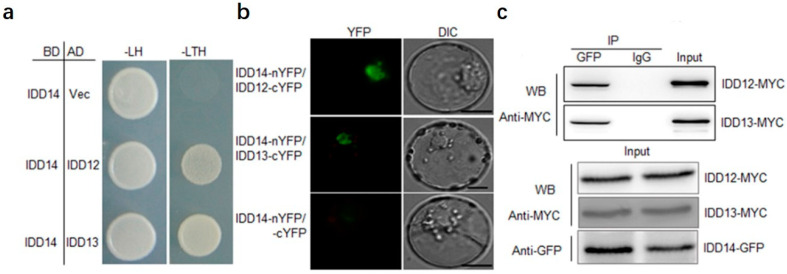
IDD14 interacts with IDD12 and IDD13: (**a**) yeast two-hybrid assay was used to test IDD14 interaction with IDD12 and IDD3. BD: GAL4-DNA binding domain; AD: activation domain; -T: without tryptophan; -L: without leucine; -H: without histidine; (**b**) BiFC assay revealed an interaction between (*IDD14* and IDD12) and (*IDD14* and IDD13) in rice protoplasts. The fused proteins of IDD14-nYFP and IDD12-cYFP, IDD14-nYFP, and IDD13-cYFP were transiently expressed in rice protoplasts. GFP fluorescence were detected in the protoplasts. IDD14-nYFP + cYFP were also coexpressed as negative control. Bars = 10 μm; (**c**) Co-IP assay was performed to analyze the interaction between IDD14 and IDD12 or IDD13 in tobacco leaves. IDD12-MYC or IDD13-MYC + IDD14-GFP were transformed into tobacco leaves by using agrobacterium-mediated transformation. Anti-GFP antibody immunoprecipitated proteins were analyzed using Western blot analysis by using anti-MYC antibodies. IDD12-MYC, IDD13-MYC, and IDD14-GFP levels were analyzed using Western blot.

The interaction between three IDDs was analyzed. The Y2H assay showed that IDD14 interacts with IDD12 and IDD13 (Figure 3a), and BiFC indicated that IDD14 interacts with IDD12 and IDD13 at the nucleus in the rice protoplasts (Figure 3b). In Co-IP, IDD14-GFP was coexpressed with IDD12-MYC or IDD13-MYC in tobacco leaves and immunoprecipitated with GFP antiserum or IgG, and the immunoprecipitated protein was immunoblotted using GFP and MYC antibodies. The results indicated that IDD14 interacts with IDD12 and IDD13 (Figure 3c). These findings suggest that IDD12, IDD13, and IDD14 may form a transcription factor complex regulating MDPK. 

### 2.4. IDD12, IDD13, and IDD14 Positively Regulate Rice Defense against ShB

**Figure 4:**
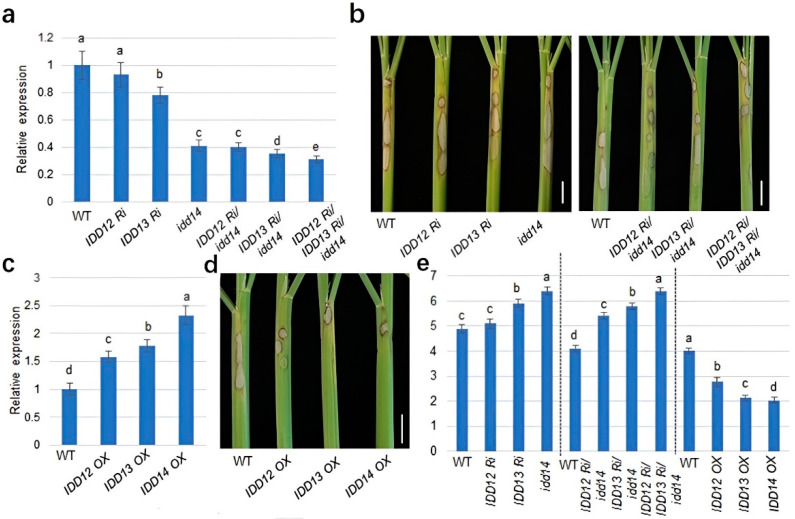
Rice ShB resistance is positively regulated by *IDD12, IDD13*, and *IDD14*: (**a**) *MDPK* expression level in wild-type, *IDD12 RNAi*, *IDD13 RNAi*, *idd14*, *IDD12 RNAi/idd14*, *IDD13 RNAi/idd14*, and *IDD12 RNAi/IDD13 RNAi/idd14* plants. *Ubiquitin* was used as reference gene to normalize gene expression levels. The error bars mean ± SE (*n* = 3); (**b**) the response of wild-type, *IDD12 RNAi*, *IDD13 RNAi*, *idd14*, *IDD12 RNAi/idd14*, *IDD13 RNAi/idd14*, and *IDD12 RNAi/IDD13 RNAi/idd14* plants to *R. solani* AG1-IA were analyzed; (**c**) *MDPK* expression level in wild-type, *IDD12 OX*, *IDD13 OX*, and *IDD14 OX* plants. *Ubiquitin* was used as reference gene to normalize gene expression levels. The error bars mean ± SE (*n* = 3); (**d**) wild-type, *IDD12 OX*, *IDD13 OX*, and *IDD14 OX* plants were inoculated with *R. solani* AG1-IA; (**e**) the lesion lengths from the plants shown in (**b**,**d**) were calculated. Data indicate average ± standard error (SE) (*n* > 8). Bonferroni’s multiple comparison test, was used for the calculation of comparisons between different groups. Letter a–e in the figure indicate significant differences at *p* < 0.05.

The *MDPK* expression levels in *IDD12*, *IDD13*, and *IDD14* single, double, and triple mutants and the overexpressing plants were investigated. The *MDPK* expression level was lower in *IDD13 RNAi* and *idd14* compared with the wild-type, while *IDD12 RNAi* and *IDD12* suppression did not affect the *MDPK* expression level. In addition, the *MDPK* expression level was much lower in *IDD12 RNAi*/*IDD13 RNAi/idd14* than in *IDD13 RNAi/idd14* and *IDD12 RNAi*/*idd14*. Meanwhile, *MDPK* expression levels were similar in *IDD12 RNAi*/*idd14* and *idd14* (Figure 4a), suggesting the additive effect of IDD12, IDD13, and IDD14 on *MDPK* expression. 

Further, *IDD12*, *IDD13*, and *IDD14* single, double, and triple mutants were inoculated with *R. solani* AG1-IA. *IDD13 RNAi* and *idd14* were more susceptible to ShB, while wild-type and *IDD12 RNAi* plants responded similarly to *R. solani*. *IDD12 RNAi*/*IDD13 RNAi/idd14* was more susceptible to ShB than *IDD13 RNAi/idd14* and *IDD12 RNAi*/*idd14* (Figure 4b,e). The qRT-PCR analysis showed that *MDPK* expression levels were higher in *IDD12 OX*, *IDD13 OX*, and *IDD14 OX* plants than in the wild-type plants (Figure 4c). The inoculation of *R. solani* AG1-IA indicated that *IDD12 OX*, *IDD13 OX*, and *IDD14 OX* plants were less susceptible to ShB than wild-type plants (Figure 4d,e). These findings suggest that IDD12, IDD13, and IDD14 positively regulate rice defense to ShB, and the lesion length negatively correlates with *MDPK* expression levels. 

### 2.5. MDPK Promotes Rice Resistance to ShB without Affecting Yield 

**Figure 5:**
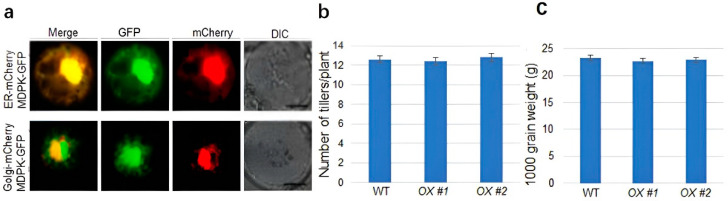
The function and subcellular localization of *MDPK*: (**a**) subcellular localization of MDPK-GFP fusion protein in rice protoplasts. Bars = 10 μm; (**b**) number of effective tillers and (**c**) 1000-grain weight per plant were calculated from wild-type and *OX #1* and *OX #2* plants. Data indicate average ± standard error (SE) (*n* > 10).

The subcellular localization was tested to predict the function of the MDPK protein. The MDPK-GFP was found colocalized with ER-mCherry and Golgi-mCherry in the rice protoplast, indicating MDPK localization in ER and Golgi (Figure 5a). Since *MDPK* overexpressors are less susceptible to ShB, the yield index was examined in *MDPK OX* plants. The results revealed that the number of effective tillers (Figure 5b) and the thousand-grain weight of *MDPK OX* plants were similar to those of the wild-type plants (Figure 5c). These findings suggest that *MDPK* overexpression promotes rice resistance to ShB without affecting the yield.

## 3. Discussion

ShB is one of the most severe diseases and a major threat to rice production. Great progress has been made in understanding the rice defense mechanism to ShB; however, the molecular mechanism behind this disease remains unclear. Therefore, the isolation of resistant cultivars and defense-related genes will be important to deepen the investigation of rice defense mechanisms. To analyze the rice defense mechanism against ShB, a previous RNA-seq-based transcriptome assay identified several rice genes differentially expressed in response to *R. solani* infection [39]. Among those genes, *MDPK* expression was significantly induced 72 h after the inoculation. The analysis of *MDPK RNAi* and *OX* plants in the present study revealed a positive correlation between the *MDPK* expression level and rice resistance to ShB. Here, *MDPK RNAi* plants were more susceptible to ShB, while *MDPK OX* plants were less susceptible compared with the wild-type. 

Furthermore, the MDPK’s upstream regulators, such as transcription factors, were screened using the Y1H assay. Interestingly, three IDD proteins, IDD12, IDD13, and IDD14, were identified via the assay. Previously, IDD14 and IDD13 were found to activate *PIN1a* and promote rice resistance to ShB. A similar regulation was expected for MDPK. The expression pattern of *IDD14* under *R. solani* infection was similar to that of *MDPK*, suggesting the role of IDD14 in regulating *MDPK* induction during infection [19,20]. Further evaluation using EMSA, ChIP, and transient assays confirmed the direct binding of IDD12, IDD13, and IDD14 to the *MDPK* promoter to activate its expression. Further analysis of the *IDD12*, *IDD13*, and *IDD14* mutants and *IDD12*, *IDD13*, and *IDD14* *OX* plants revealed that three IDDs positively regulate *MDPK* expression. *IDD12* suppression did not change *MDPK* expression, while *IDD12* overexpression upregulated *MDPK* levels, suggesting a weak effect of IDD12 on activation of *MDPK*. *IDD13* and *IDD14* mutants were more susceptible to *R. solani* infection, while the *OX* plants were less susceptible. Meanwhile, the *IDD12* mutant exhibited a symptom similar to the wild-type, while *IDD12 OX* plants were less susceptible to ShB. These observations collectively suggest that the role of IDD12, IDD13, and IDD14 in regulating *MDPK* expression was associated with disease resistance in rice plants. 

IDD14 has been known to interact with IDD13 to activate *PIN1a* expression [20]. The present study’s Y2H, BiFC, and Co-IP assays showed that IDD14 interacts with IDD12 and IDD13, which suggests that the three IDDs might form a transcription factor complex to regulate downstream gene expression. Furthermore, to verify whether the three IDDs have functional redundancy on the activation of *MDPK*, a transient assay was performed by coexpressing the IDDs. The coexpression of the three IDDs demonstrated the highest effect on the activation of *MDPK*. Meanwhile, IDD13 + IDD14 exhibited higher activation activity than IDD12 + IDD13 or IDD12 + IDD14, indicating the minimal role of IDD12 in *MDPK* activation; however, the three IDDs together had a synergistic effect. The analysis of the mutants revealed the lowest *MDPK* expression level in *IDD12 Ri/IDD13 Ri/idd14* triple mutants compared with *IDD12 Ri/idd14* or *IDD13 Ri/idd14*. A subsequent inoculation of *R. solani* showed that the symptoms in *IDD12 Ri/IDD13 Ri/idd14* plants were more severe than in *IDD12 Ri/idd14* or *IDD13 Ri/idd14* double mutants. These data suggest that IDD13 and IDD14 play crucial roles in activating *MDPK*, while IDD12 plays a weak role. 

MDPK homologs have been reported to play key roles in arbuscular mycorrhizal (AM) symbiosis in rice [40] and immune response in barley [31] and *Arabidopsis* [32]. These studies suggested the potential function of MDPK in plants and microbe interaction. In the present study, the MPDK-GFP was found colocalized with ER and Golgi markers, suggesting the role of MDPK in protein maturation and secretion. Moreover, several studies have predicted MDPK as a kinase; therefore, further analysis is required to identify its potential substrates and clarify its function in plant–microbe interaction. 

Generally, the high yield cultivars exhibit susceptible symptoms due to the antagonistic relationship between crop yield and immunity pathways [41]. The *IDD13* and *IDD14* *OX* plants generated in this study demonstrated high ShB resistance without any yield reduction [19,20]. The effective tillers and thousand-grain weight of the *MDPK OX* plants were similar to those of the wild-type. These observations suggest that the *MDPK OX* plants, similar to *IDD13 OX* and *IDD14 OX* plants, enhance rice resistance to ShB with no yield loss. Overall, these results broaden our understanding of the IDD-mediated mechanism and MDPK function in ShB defense. The study also proposes a useful marker for ShB resistance breeding. 

## 4. Materials and Methods

### 4.1. Plant Cultivation and R. solani Inoculation 

The wild-type (WT) control line (*O. sativa Japonica*, cultivar Dongjin), *MDPK* RNAi and overexpressing plants; *IDD12*, *IDD13*, *IDD14* single, double, triple mutants and the overexpressing plants were used. Three independent biological replicates with 10 mature rice seeds per replicate were surface sterilized with 2% sodium hypochlorite (NaClO) and rinsed three times with sterile water. The sterilized seeds were wrapped in a wet towel and placed in an incubator (37 °C, dark) for 2–3 days for germination. The germinated seeds were placed in a floating tray containing soil for further growth. Veneers were cut into 1 cm × 0.5 cm size pieces and placed on potato dextrose agar (PDA) plates inoculated with *R. solani* AG1-IA in the middle. These PDA plates were maintained in an incubator at 28 °C under dark. The veneers covered with mycelium were later inserted into the first sheath of the one-month-old rice seedlings, and the inoculated sheath was wrapped with a cling film. The lesion length was measured after 10–14 days of inoculation. 

### 4.2. Plasmid Construction and Rice Transformation 

To generate the overexpression constructs, the ORF of *MDPK, IDD12*, *IDD13*, and *IDD14* were amplified and then cloned into a pCAMBIA1302 binary vector with a CaMV 35S promoter. To generate *MDPK* RNAi plants, 300 bp of the *MDPK* coding region was cloned into SwaI and AscI sites in the sense and XbaI and BamHI sites in the antisense orientation, respectively, in the pFGC5941 binary vector (ChromDB). Then, the above constructs were transformed into cultivar Dongjin by the agrobacterium (*Agrobacterium tumefaciens*)-mediated rice mature embryo transformation method.

### 4.3. Real-Time Quantitative PCR (qRT-PCR) 

Total RNA was extracted from the rice plants using a TRIzol reagent (Takara, Dalian, China). DNA removal and cDNA synthesis were performed using a PrimeScript RT reagent Kit (Takara, China), following the manufacturer’s instructions. Subsequently, a qRT-PCR assay was performed using Ssofast EvaGreen Supermix (BIO-RAD, Hercules, CA, USA) on an Mx3005P system (Agilent, Santa Clara, CA, USA) using rice ubiquitin (LOC_Os03g13170) as the internal reference gene [42]. Three technical replicates were maintained per treatment. The primers used for the qRT-PCR are listed in Table S1.

### 4.4. Yeast One-Hybrid (Y1H) Assay and cDNA Library Construction

Total RNA was extracted from 12-day-old young rice seedlings to construct the cDNA library for isolating the regulator of *MDPK* using Matchmaker Gold Systems (Clontech, Dalian, China), following the manufacturer’s instructions. About 2 kb long *MDPK* promoter was cloned into a pHISi-1 vector. Finally, IDD12, IDD13, and IDD14 genes were selected for further confirmation. The ORF sequences of *IDD12*, *IDD13*, and *IDD14* were cloned into a pGAD424 vector. Then, the *pHISi-1-pMDPK* and *pGAD424-IDD12/13/14* vectors or *pGAD424* empty vector (negative control) were transformed into the yeast strain YM4271. The transformed yeasts were selected on SD-Leu or SD-His plates with 3-amino-1,2,4-triazole (3AT). 

### 4.5. Electrophoretic Mobility Shift Assay (EMSA)

The ORFs of *IDD12/13/14* were cloned into a pET28a(+) vector to produce the recombinant proteins. Further, an electrophoretic mobility shift assay (EMSA) was performed as previously described [43]. In this assay, 1 µg of His:IDD12/13/14 protein and a biotin-labeled DNA probe were used. The probe was generated using an EMSA/Gel-Shift kit (Beyotime, Shanghai, China), following the manufacturer’s instructions. 

### 4.6. Transactivation Activity Assay

Rice protoplast was cotransformed with the *pMDPK-GUS* reporter and the *35S*:*IDD12*/*13*/*14* effector [44], using *35S:LUC* as the internal control. The GUS expression of the transformed protoplasts was normalized against luciferase expression [45]. Transformation and transactivation activity assays were carried out as reported earlier [46]. 

### 4.7. Chromatin Immunoprecipitation (ChIP) Assay

Two grams of *IDD12/13/14-GFP* transgenic plants grown for two weeks were collected for ChIP assay. The GFP monoclonal antibody and the pre-immune serum (IgG) (Abcam, England, UK) were used for immunoprecipitation after pre-absorption with the IgG. The DNA was eluted, subjected to reverse crosslinking, and used for PCR analysis. The corresponding input DNA was used as an internal reference in immunoprecipitation [43].

### 4.8. Yeast Two-Hybrid (Y2H) Assay 

The complete ORF of *IDD14* was cloned into a pGBT9 vector, and the ORFs of *IDD12/13* were cloned into a pGADT7 vector. The pGBT9-IDD14 and pGADT7-IDD12/13 vectors or the pGADT7 empty vector (negative control) were cotransformed into the yeast strain PJ69-4A. The transformants were selected on the SD-Leu/SD-His(-LH) and SD-Trp/-Leu/-His (-TLH) plates (-T: without tryptophan; -L: without leucine; -H: without histidine).

### 4.9. Bimolecular Fluorescence Complementation (BiFC) Assay

IDD14 was fused to the N-terminal of the yellow fluorescent protein (YFP), and IDD12/13 was fused to the C-terminal of YFP in the PU-nYFP and PU-cYFP plasmids, respectively, modified from a PU19 vector. For BiFC assay, the IDD14-nYFP and IDD12/13-cYFP plasmids or the cYFP plasmid (negative control) were transformed into rice protoplasts, following a previously reported method [46]. Fluorescence of rice protoplasts was observed under a fluorescence microscope Olympus X1000 (Tokyo, Japan).

### 4.10. Coimmunoprecipitation (Co-IP)

The IDD14-GFP and IDD12-MYC or IDD13-MYC plasmids were cotransformed into tobacco leaves for transient expression. The immunoprecipitated protein was immunoblotted using green fluorescent protein (GFP) and MYC antibodies. The protein extraction and Co-IP assay were performed as previously described [47].

### 4.11. Subcellular Localization

For subcellular localization of MDPK, the ORF of *MDPK* was cloned into a PU19-GFP vector. The MDPK-GFP plasmid was cotransformed with ER-mCherry or Golgi-mCherry plasmids into the rice protoplast [46]. The fluorescence of the rice protoplasts was observed under a fluorescence microscope (Olympus X1000).

### 4.12. Statistical Analysis

Quantification analyses were performed regarding the relative expression level of different genes, effective tiller numbers, thousand-grain weight, lesion length, and GUS activity. Prism 8 (GraphPad, San Diego, CA, USA) was used for statistical analysis. One-way analysis of variance (ANOVA), followed by Bonferroni’s multiple comparison test, was used for the calculation of comparisons between different groups. Data indicate average ± standard error (SE). The different letters indicate significant differences at *p* < 0.05. Detailed descriptions of quantifications and statistical analyses can be found in the figures and figure legends.

## Data Availability

The data presented in this study are available on request from the corresponding author.

## Supplementary Materials

The following supporting information can be downloaded at: https://www.mdpi.com/article/10.3390/ijms23158214/s1.
