# Supplementary material for: Malectin Domain Protein Kinase (MDPK) Promotes Rice Resistance to Sheath Blight via IDD12, IDD13, and IDD14
Source: Int J Mol Sci. 2022 Jul 26;23(15):8214. doi: 10.3390/ijms23158214 (PMC9331740; doi:10.3390/ijms23158214)
Supplement: Supplementary file 1 [file ijms-23-08214-s001.zip › ijms-1809105-supplementary.pdf]

Supplemental Table

Table S1. Primers used in this study

pMDPK-F	AAGCTTGTAGGCGAGAACAGCAGTCAG
pMDPK-R	GGATCCCCAAGATCCTCGCTTATTTAC
MDPK-F	AAGCTTATGCCCTGCAGTCCGCCATC
MDPK-R	GGTACCCCTCGCTGTTGGCATGGAGTT
IDD12-F	AAGCTTATGCTGAGTTCTTGCGCGCCGAC
IDD12-R	GGTACCGTTGAGGTCCATTGCCATCGTCCTAG
IDD13-F	AAGCTTATGTTGGGTTCTTGCGCCCCGA
IDD13-R	GGTACCCATGATGCCCATGCTGTTAGCGTGCTG
IDD14-F	ATGGCACTGGTCAAGAGCCAC
IDD14-R	GATGCCGGCGCCGACGCCGCCG
IDD12 RT-F	GATCAGCTCCGACGTCGCCA
IDD13 RT-F	CGCCACTACCGGCGCCATG
IDD14 RT-F	GCGCGCCTGATCGCGCAGGTG
MDPK RT-F	TAGTGAAAGGCCAACGATGAG
P1-F	AGCAGTCAGTGGCGACAAAGGCATGCTGGTG
P1-R	CACCAGCATGCCTTTGTCGCCACTGACTGCT
mP1-F	AGCAGTCAGTGGAAAAAAAGGCATGCTGGTG
mP1-R	CACCAGCATGCCTTTTTTTCCACTGACTGCT
Ubiquitin-F	CAAGATGATCTGCCGCAAATGC
Ubiquitin-R	TTTAACCAGTCCATGAACCCG
